# Abrotanum: With Clinical Cases

**Published:** 1888-02

**Authors:** J. T. Kent

**Affiliations:** St. Louis


### ABROTANUM: WITH CLINICAL CASES.

Professor J. T. Kent, M. D., St. Louis.

Irritable, week-minded, worse from mental exertion.

The head topples over because the neck is emaciated: the face is wrinkled and has a sickly look: the temples are marked by distended veins.

The face looks old, the infant looks like a little old person. [Also, Bar., Calc., Iodine, Natr-m., Op., Sulph.] [If from syphilis, Aur-mur.]

The whole body is emaciated and wrinkled; the emaciation spreads from the lower limbs upward (which is the reverse of Lyc. and Natr-m.)

Enlarged glands, especially in the emaciated abdomen.

Diseases change from place to place [metastasis]. Mumps go to the mammæ or to the testes. Rheumatism leaves the joints and endocarditis appears with profuse sweat; cannot lie down for the dyspnœa; sinking as if dying, pulse feeble. Rheumatism comes on when a diarrhœa has ceased too suddenly.

Piles which get worse as the rheumatism abates. Bleeding from the piles in amenorrhœa. [Graph.]

Hydrocele in boys.

Distended abdomen. [Ars., Bar., Calc., Iodine, Lyc., Puls., Sulph.]

Piercing pains in the heart. Piercing in the ovaries, mostly the left.

Wakes in a fright and trembles, is covered with cold sweat.

The extremities are numb and tingle as if thawing, after having been frozen.

High fever after the rheumatism has gone to the heart.

The wasting child has hectic fever with a ravenous appetite. Lives well yet emaciates. [Also Iodine, Natr-m.]

Abrotanum attacks the white fibrous tissues, the joints, pleura, peritoneum, etc.

Gouty nodosities in the wrist and the fingers.

Rheumatism goes to the heart, compare with Cactus, Dig., Kalmia, Lach., Naja, Spig., Spongia.

The grand features of this remedy are: metastasis; marasmus spreading upward.

Case 1. Mrs. P. suffered from gouty deposits about the finger joints, which were very painful during cold, stormy weather. The joints and nodes were sore and hot at such times. The nodes ceased to be painful and sudden hoarseness came; ulcers in the larynx followed; great dryness in the nose and painful dry throat; sticking in the cardiac region. She lost flesh but the appetite kept good. Calc-phos. had been prescribed by her former attendant. After duly considering the case, Abrot.$^{45m}$ [F.] was given. She suffered for many days after this dose with a most copious discharge from her nose and bronchial tubes; expectoration was copious, thick, yellow. Hoarseness ceased at once. In a month she ceased coughing; the finger joints became painful and swollen considerably. In three months she had no pain and the nodes were scarcely perceptible.

She is now perfectly well and has been so one year. She had only one dose of the remedy, as the case was doing well enough, *i. e.*, as the symptoms were taking the right course to recovery in the proper way. She suffered much pain on the road to recovery but I know of only one way to cure these cases, and that is to let the remedy alone when the symptoms are taking the proper course.

Case 2. Mrs. T. had suffered from chronic rheumatism of the left ankle and knee for several years. She rubbed the limb with a strong liniment and the rheumatism was speedily cured. But it was not long before she needed a physician. I saw her with friends surrounding her bed, she was covered with a profuse, cold sweat, sitting propped up on pillows. Her friends said she was dying, and I thought so too. She had a small, quick pulse; there was pain at the heart and auscultation over heart, revealed the usual story, which is too well known to all, as there are many such cases. She was six months pregnant. Gave her Abrot., and she slowly recovered. The little one now bears my Christian name in honor of the great cure. She has recovered, perfectly free from rheumatism, and the lad is now several years old.

These two cases show what Abrotanum can do when properly indicated. It is a powerful remedy and must not be repeated. It acts many weeks, in waves or cycles; it is too seldom used.

